# Safety and feasibility of open abdomen with negative pressure therapy in major liver trauma: a retrospective bicenter study

**DOI:** 10.1007/s00423-025-03729-1

**Published:** 2025-05-16

**Authors:** Andrea Coppola, Arianna Mottola, Marcello Della Corte, Mariafelicia Valeriani, Giovanni Aprea, Patrizio Festa, Giuseppe Palomba

**Affiliations:** 1 Azienda Ospedaliera Universitaria San Giovanni di Dio e Ruggi d’Aragona, Salerno, 84131 Italy; 2 Trauma Center, “Antonio Cardarelli” Hospital, Via A. Cardarelli 9, Naples, 80131 Italy; 3 Department of Clinical Medicine and Surgery, University of Naples, “Federico II”, Sergio Pansini 5, Naples, 80131 Italy

**Keywords:** Open abdomen, Liver injury, Negative Pressure Therapy, Perihepatic packing, Damage control surgery, Abdominal trauma

## Abstract

**Background:**

Abdominal trauma is the third leading cause of death in the young population, and liver trauma is among the most common. For major liver injury, perihepatic packing (PHP) is a life-saving procedure that allows rapid control of the hemorrhage. The use of the open abdomen (OA) represents a strategy for the management of major abdominal trauma. However, the effects of combined use with Negative Pressure Therapy (NPT) in patients with liver trauma are not yet clear. The aim of our study was to evaluate the safety and feasibility of OA with NPT.

**Materials and methods:**

This retrospective bicenter study enrolled all patients affected by liver trauma who underwent operative management from January 2019 to September 2023 at the Emergency Surgical Units of the “A. Cardarelli” Hospital in Naples and the “San Giovanni di Dio e Ruggi d’Aragona” in Salerno. The two groups were compared in terms of intra- and postoperative outcomes.

**Results:**

Out of a total of 213 patients with liver trauma, 43 patients were divided into two groups: group A (24 patients treated with PHP and OA) and group B (19 patients with PHP, OA and NPT). There were no significant differences in terms of demographic data, preoperative characteristics, length of stay, mortality, or intraoperative or postoperative complications. Hb increase at the time of depacking was higher in group B (*p* = 0.039).

**Conclusions:**

Open Abdomen with Negative Pressure Therapy appears safe and feasible. Furthermore, it does not affect mortality or hospital stay but it seems to be associated with higher hemoglobin (Hb) levels during the depacking phase.

## Introduction

Trauma is the leading cause of death in the young population and is associated with high morbidity and mortality [1]. Abdominal trauma is the third most common type of trauma, and the liver is the most frequently injured abdominal organ [2].

The management of liver trauma requires a multidisciplinary approach [3]. Using the Organ Injury Scale of the American Association for the Surgery of Trauma (AAST-OIS) and on the basis of the hemodynamic status of patients, liver injuries are categorized into four grades of increased severity by the World Society of Emergency Surgery (WSES) [3].

In the last four decades, the treatment of minor to moderate injuries (WSES I, II, III) (AAST-OIS I, II, or III) has shifted, in selected patients, to nonoperative management (NOM), even when active bleeding is present on computerized tomography, due to advances in angiography/angioembolization (AG/AE) and endoscopic treatments, leading to a reduction in hospital mortality and morbidity [2–6].

It has been estimated that the NOM of liver injuries ranges from 50 to 85% [2] and can safely be the treatment of choice in both level 1 and level 2 trauma centers [3, 4].

Conversely, operative management (OM) remains the treatment of choice for hemodynamically unstable patients and nonresponders (WSES IV) [3]. Despite technical and therapeutic advances, mortality in these patients remains high [2, 3].

To reduce perioperative mortality, damage control surgery (DCS) is currently the treatment of choice for hemodynamically unstable trauma patients, leaving the definitive surgical treatment of all injuries to be performed at a later stage [7]. For major liver trauma, in the case of massive bleeding, perihepatic packing (PHP) is a life-saving procedure that allows rapid control of the hemorrhage and fast access to the intensive care unit (ICU) [3, 8, 9]. However, PHP is not without problems, with the main complications being rebleeding at the time of packing removal (depacking), liver necrosis and abdominal compartment syndrome [10].

Since the 1990s, the practice of open abdomen (OA) has been an integral part of the management of severe abdominal trauma in trauma patients [11–14]. The WSES guidelines on OA in trauma and nontrauma cases highlight its effectiveness in counteracting the imbalanced physiology typically present in critically ill or severely injured individuals [13]. The development of enteroatmospheric fistulas (EAFs), “frozen abdomen” (FA), abdominal compartment syndrome (ACS), intra-abdominal abscesses and fascial retraction are the main complications of this technique [13, 15].

In recent years, temporary abdominal closure (TAC) associated with negative pressure therapy (NPT) has been proposed for trauma patients [16]. This method allows the reduction of intestinal edema and the onset of abdominal hypertension. It is also believed to improve local blood perfusion by increasing the formation of granulation tissue [16, 17]. Furthermore, NPT with fascial traction shows better results in terms of delayed abdominal closure, incisional hernia, EAF and the enterocutaneous fistula rate [13, 16]. The use of NPT in combination with the instillation of fluids for TAC appears to improve outcomes in trauma patients, but its real merits are still being debated [13, 18].

To our knowledge, our study is the first to evaluate the use of OA with NPT in trauma patients treated with liver PHP. The aim of our study was to compare PHP associated with OA and NPT versus PHP alone and/or OA without NPT in patients with major liver trauma in terms of intra- and postoperative outcomes.

## Materials and methods

This retrospective bicenter study enrolled all patients affected by liver injury who underwent OM from January 2019 to September 2023 at the Emergency Surgical Units of the “A. Cardarelli” Hospital in Naples and the “San Giovanni di Dio e Ruggi d’Aragona” in Salerno.

Data were collected from the respective hospital databases. The following exclusion criteria were applied: body mass index (BMI) ≥ 40, pregnancy, intraoperative death, Glasgow Coma Scale (GCS) score ≤ 8, and age over 75 and under 18 years.

Patients were divided into two groups: group A, which included patients treated with PHP and OA, and group B, which included patients treated with PHP and OA with NPT. The demographic characteristics considered were age, sex and BMI.

The following perioperative outcomes were analyzed: grade of liver injury (according to the WSES and AAST-OIS classification), Injury Severity Score (ISS), Heart Rate (bpm), Systolic Blood Pressure (mmHg), Glasgow Coma Scale (GCS), Hemoglobin (Hb, g/dl), pH, Lactate (mmol/l), International Normalized Ratio (INR), Base Excess (B.E., mol/l), Alanine aminotransferase (ALT, U/L), Aspartate aminotrasferase (AST, U/L) before surgery and at the time of depacking, number of gauzes used, operative time (minutes), total duration of negative pressure (minutes), total number of surgeries, intraoperative embolization, use of inotropes, units of packed red cell transfusions, intraoperative or postoperative complications, length of stay (days), mortality within 7 days and within 30 days.

All patients underwent emergency laparotomy, Pringle maneuver, complete liver mobilization and subsequent placement of an adequate number of gauze dressings, together with standardized actions to control bleeding, including an effective fluid resuscitation/transfusion protocol and careful intensive care management. While the abdominal wall was left open without NPT in group A patients, the open abdomen technique with NPT (Abthera™ device, KCI, San Antonio, Texas) was used in group B patients. We used 3 M™ AbThera™ Advance Open Abdomen Dressing Components with Fenestrated Visceral Protective Layer and Advance Perforated Foam (KCI, San Antonio, Texas). The mean negative pressure was 78.53 mmHg, with a mean overall duration of 53.7 h for less than 7 days. The first dressing change after the index laparotomy took place at the time of depacking, and thereafter at 48 h intervals.

For statistical analysis, comparisons of categorical data between the two groups were performed via the χ2 test with Yates’ correction or Fisher’s exact test, when appropriate. Categorical data are reported as frequencies and percentages, whereas continuous variables are reported as means ± SDs (ranges). *P* < 0.05 was considered statistically significant. Statistical analysis was performed via IBM SPSS Statistics for Windows, version 20.0. IBM SPSS Statistics version 26 (SPSS Inc. Chicago, IL, USA) was used.

The study was performed in accordance with the principles of the Declaration of Helsinki and its appendices. Approval from Health Management, Institutional Review Board and Ethics Committee was obtained.

## Results

**Fig. 1:**
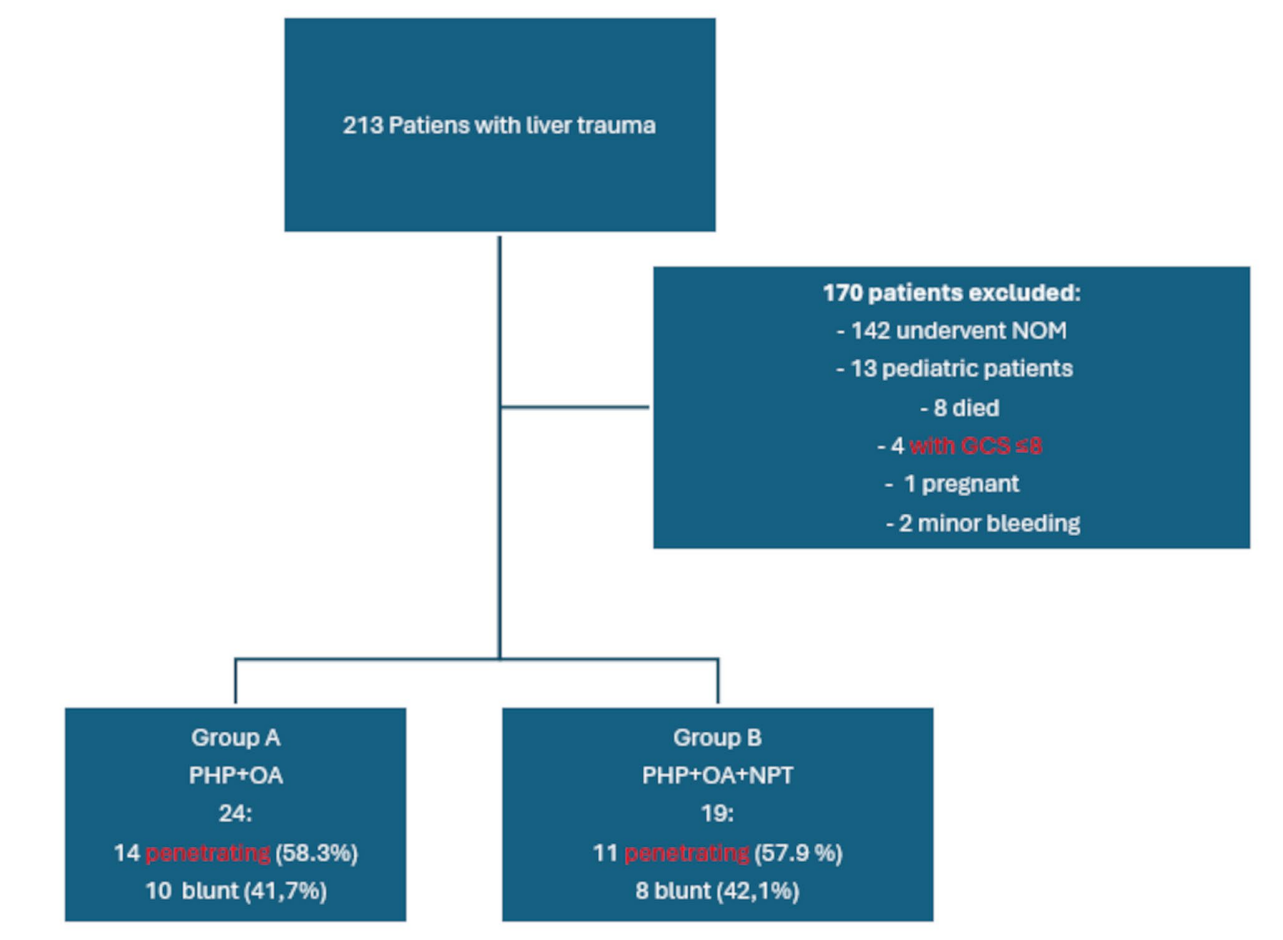
Flowchart of patient selection

Out of a total of 213 patients with liver trauma, 43 patients were divided into two groups: group A, 24 patients were treated with PHP and OA, and group B, 19 patients with PHP, OA and NPT. A total of 170 patients were excluded, of whom 142 underwent NOM, 13 were pediatric patients, 8 died before or during surgery, 4 with GCS ≤ 8, 1 was pregnant, and 2 had minor bleeding requiring simple hemostasis (Fig. 1). The demographic characteristics (age, sex, BMI, ISS, extra abdominal injuries, Heart Rate on admission Systolic Blood Pressure on admission, GCS and grade of injury) are shown in Table 1, and for these variables, there were no statistically significant differences. Associated extra-abdominal injuries in group A include four extremity injuries, eleven chest injuries, with seven pneumothoraces, three head and neck injuries, whereas in group B there were five extremity injuries, four chest injuries with three pneumothoraces and two head traumas.

**Table 1:** Characteristics of injured patients assigned to two of the groups. BMI: body mass index; ISS: injury severity score; GCS: Glasgow coma scale; SBP: systolic blood pressure; PHP: perihepatic packing; OA: open abdomen; NPT: negative pressure therapy

	Group A (24) PHP + OA	Group B (19) PHP + OA + NPT	P
Age, years	31,2 ± 8,9	30,9 ± 8,2	0.92
Sex (M/F)	16/8	13/6	0,83
BMI, kg/m2	25.48 ± 4,3	26.15 ± 4.64	0.61
Grade of injury (AAST-OIS):			
- Grade III	3	3	0.75
- Grade IV	13	11	0.8
- Grade V	8	5	0.61
ISS	23,9	23,8	0,9
Extra abdominal injuries	16	10	0,34
Heart Rate (bpm) on admission	101 ± 13,6	105 ± 12,9	0,97
SBP (mmHg) on admission	97 ± 15,5	95 ± 16,5	0,81
GCS	12,4 ± 1,82	12,7 ± 1,66	0,6

Tables 2 and 3 show the pre- and postoperative laboratory variables, respectively. The Hb concentration at the time of depacking was significantly lower in patients in Group A (9,41 ± 1,60 versus Group B (10,56 ± 1,26, *p* = 0.039). The trend of Hb within the individual groups before surgery and at the time of depacking was analysed. A greater increase in Hb levels was observed in group B, even though it did not reach statistical significance, by a narrow margin (Table 4).

**Table 2:** Preoperative blood test results in the two groups of patients. Hb: hemoglobin; INR: international normalized ratio; B.E: base excess; AST: aspartate aminotransferase; ALT: alanine aminotransferase

	Group A	Group B	*P*
pH	7,28 ± 0,10	7,3 ± 0,10	0,73
Lactate (mmol/l)	3,78 ± 2,70	3,9 ± 1,26	0,71
Hb (g/dl)	9,5 ± 2,24	9,88 ± 1,81	0,57
INR	1,35 ± 1,19	1,33 ± 0,22	0,32
B.E. (mol/l)	-3,05 ± 4,69	-3,62 ± 3,93	0,71
AST (U/L)	676,5 ± 749,3	872,3 ± 845,1	0,51
ALT (U/L)	882,4 ± 989,9	1048,1 ± 1007,2	0,37

**Table 3:** Blood test results at depacking. Hb: hemoglobin; INR: international normalized ratio; B.E: base excess; AST: aspartate aminotransferase; ALT: alanine aminotransferase

	Group A	Group B	*P*
pH	7,37 ± 0,10	7,39 ± 0,04	0,42
Lactate (mmol/l)	1,85 ± 1,33	2,33 ± 2,21	0,50
Hb (g/dl)	9,41 ± 1,60	10,56 ± 1,26	**0**,**039**
INR	1,45 ± 0,59	1,33 ± 0,56	0,61
B.E. (mol/l)	0,45 ± 7,3	3,6 ± 5,9	0,15
AST (U/L)	895,3 ± 989,1	807,1 ± 907,4	0,79
ALT (U/L)	1453 ± 1271	1092 ± 1037	0,52

**Table 4:** Comparison between hb levels preoperatively and at the time of depacking within individual groups

	Preoperative	At depacking	*p*
Group B - Hb (g/dl)	9,88 ± 1,81	10,56 ± 1,26	0,06
Group A - Hb (g/dl)	9,5 ± 2,24	9,41 ± 1,60	0,94

There were no statistically significant differences between the two groups in terms of intraoperative or postoperative variables (Table 5). Intraoperative complications included bleeding, which was treated at the time of surgery (6 versus 3, *p* = 0,46). There were 14 postoperative complications in group A and 9 in group B (*p* = 0.49). In group A, 4 biliary fistulas and 2 bilomas were treated conservatively: 2 abscesses, 1 case of late renal lodge bleeding after depacking, 1 case of evisceration and 1 case of deep vein thrombosis. One of the patients with biliary fistula developed pneumothorax on the second postoperative day, which was treated with a chest drain; two patients also developed pneumonia and were treated with standard medical therapy. In Group B, there was 1 case of liver necrosis resulting in septic shock and death, 3 cases of biliary fistula and 1 case of biloma, one patient who presented with both EAF and FA, 1 right colon ischemia that was surgically treated, 1 case of late bleeding and 1 ischemic arm. Two patients presented with pneumothorax treated with a chest drain, and two also presented with pneumonia treated with standard medical therapy.

**Table 5:** Intraoperative and postoperative variables

	Group A	Group B	*P*
Number of gauzes used for packing	5,7 ± 2,1	5,23 ± 1,43	0,57
Operative time	95,7 ± 34	101,6 ± 23	0,51
Perioperative use of inotropes	9 (37,5%)	7 (36,84%)	0,96
Number of transfusions (mean)	3,3 ± 0,8	2,9 ± 0,7	0,73
Intraoperative embolization	7 (29,17%)	5 (26,32%)	0,83
Intraoperative complication	6 (25%)	3 (15,8%)	0,46
Postoperative complication	14 (58,3%)	13 (68,4%)	0,49
Length of stay	25,72 ± 16,65	43,33 ± 34,5	0,063
Number of surgeries	3 ± 2,4	2,4 ± 0,64	0,25
Mortality within 7 days	3 (12,5%)	2 (10,53%)	0,84
Mortality within 30 days	2 (8,3%)	3 (15,8%)	0,44

All patients were transfused, and there was no difference between the two groups.

In group 2, the mean negative pressure was − 78.53 mmHg, with a mean overall duration of 53.7 h. Five major liver resections were required in group A, and 3 major liver resections were required in group B. In group A, in addition to PHP, splenectomies were performed in three patients. In group B, two splenectomies were performed. At the time of depacking, two splenectomies and two nephrectomies were required in group A, whereas in group B, one splenectomy, one right colon hemicolectomy, an ileal resection and two pancreaticoduodenectomy (PD) were necessary.

Finally, there were no statistically significant differences in terms of mortality at 7 and 30 days between the two groups.

## Discussion

The management of liver trauma represents one of the greatest challenges in trauma centers and surgical units, both for NOM and OM [3]. In hemodynamically unstable patients, OM remains the only therapeutic choice available. PHP is the most effective technique for preventing bleeding in severe liver trauma patients [9, 19–23]. However, if not correctly performed, this procedure may not achieve adequate control of the hemorrhage or lead to overpacking complications such as compartment syndrome, reduced venous return, worsening hemodynamic status and liver distress [9, 10, 24].

Proper management of abdominal wall closure in DCS is essential in situations where there is a high risk of ACS and the need for a second look [7]. Over the last decade, OA with and without NPT has been shown to be safe and effective in preventing ACS, a frequent event after abdominal trauma [25–27].

The use of NPT minimizes fascial retraction and loss of domain and allows drainage of intraperitoneal fluids, counteracting the onset of ACS [25–27]. EAF and FA can be possible complications of OA with NPT [28, 29]. To prevent the onset of complications such as inability to close, incisional hernias, evisceration and EF, fascial closure should be performed within seven days of the first intervention [13, 30, 31]. In our study, one case of evisceration occurred in group A, and one EAF developed in group B, resulting in prolonged hospital stay.

Liver trauma is the result of high-energy trauma and often involves multiple body areas, affecting patient prognosis and treatment. The length of stay in patients with liver trauma is also strongly affected by the presence of concomitant injuries. In our study, the trend showed a longer hospital stay in group B; however, the difference between the two groups was, by a narrow margin, not statistically significant (*p* = 0.063). Patients with liver injuries in which more than one other organ seemed affected at the time of the index operation were excluded to reduce the effect of confounding factors. The presence of highly complex lesions related to reconstructive difficulty should be considered in the decision-making process. In group B, two duodenal lesions of AAST-OIS grade II and III required PD, and multiple reconstructive operations. One of these patients had both EAF and AF, resulting in a long hospital stay.

Although negative-pressure OA is a safe and effective treatment for abdominal trauma, to reduce the risk of hemorrhage due to both injury and coagulopathy, adequate pressure values ​​must be used. In accordance with international recommendations, we used a mean negative pressure of -78.53 mmHg, with a mean overall duration of 53.7 h for less than 7 days [29, 32].

No significant differences were found between the two groups in laboratory parameters before and after depacking, with the sole exception of Hb, which in group B was higher at the time of depacking in group B. This result might be due to the systemic anti-inflammatory and vasoconstrictive effects of negative pressure. Regarding the number of major liver resections, only three major resections had to be performed in Group B, whereas five in Group A, for the same degree of lesions. However, the difference was not statistically significant.

These data could be linked to the local and systemic improvement induced by the reduction in proinflammatory cytokines and the release of angiogenic factors. G. E. Glass et al. demonstrated that negative pressure improves the inflammatory and interleukin response induced by trauma [33]. Aspiration and drainage of posttraumatic peritoneal fluid reduce the blood and local levels of proinflammatory cytokines, with important transduction of mechanoreceptors and chemoreceptors for angiogenesis and hematopoiesis [33]. These mechanisms could improve local and medullary conditions at molecular level, promoting faster healing [33].

Regarding the need for inotropes, transfusions, embolization and mortality at 7 and 30 days, there were no differences between the two groups, further confirming the overlap of the two techniques.

There are few studies on the use of OA with NPT in trauma patients. There is unanimous consensus that NPT results in a high rate of fascial closure and low complication rates in these patients [30, 31, 34–36]. To our knowledge, this is the first study investigating the role of PHP and OA with NPT in patients with liver trauma.

This study has several limitations. The most important is the retrospective nature of the study and the nonrandomization of the groups, with potential biases such as patient selection and the choice of surgical procedure. The number of enrolled patients is not high because major liver trauma is highly fatal, is often associated with injuries to various anatomical districts, and during the lockdown period (which began in March 2020 with a gradual easing of restrictive measures beginning in May 2020), there was a reduction in hospitalizations and emergency surgical interventions in our units [37, 38]. Therefore, randomized comparative studies and multicenter studies are needed to further evaluate the real benefits of NPT combined with OA in patients with liver trauma.

## Conclusions

Based on preliminary data from our study, in patients with liver trauma undergoing perihepatic packing and open abdomen, negative pressure therapy appears safe and feasible even compared to perihepatic packing alone. Furthermore, it seems to be associated with higher Hb levels during the depacking phase. Since our analysis is a proof of concept, randomized and multicenter studies are needed to confirm our results.

## Data Availability

No datasets were generated or analysed during the current study.
